# *BIIDXI*, the *At4g32460* DUF642 gene, is involved in pectin methyl esterase regulation during *Arabidopsis thaliana* seed germination and plant development

**DOI:** 10.1186/s12870-014-0338-8

**Published:** 2014-12-02

**Authors:** Esther Zúñiga-Sánchez, Diana Soriano, Eleazar Martínez-Barajas, Alma Orozco-Segovia, Alicia Gamboa-deBuen

**Affiliations:** Instituto de Ecología, Universidad Nacional Autónoma de México, Apartado Postal 70-275, Ciudad Universitaria, México, 04510 Distrito Federal Mexico; Facultad de Química, Universidad Nacional Autónoma de México, Ciudad Universitaria, México, 04510 Distrito Federal Mexico

**Keywords:** DUF642 proteins, Pectin methyl esterases, Germination

## Abstract

**Background:**

DUF642 proteins constitute a highly conserved family of proteins that are associated with the cell wall and are specific to spermatophytes. Transcriptome studies have suggested that members of this family are involved in seed development and germination processes. Previous in vitro studies have revealed that *At4g32460-* and *At5g11420*-encoded proteins interact with the catalytic domain of pectin methyl esterase 3 (AtPME3, which is encoded by *At3g14310*). PMEs play an important role in plant development, including seed germination. The aim of this study was to evaluate the function of the DUF642 gene *At4g32460* during seed germination and plant development and to determine its relation to PME activity regulation.

**Results:**

Our results indicated that the DUF642 proteins encoded by *At4g32460* and *At5g11420* could be positive regulators of PME activity during several developmental processes. Transgenic lines overexpressing these proteins showed increased PME activity during seed germination, and improved seed germination performance. In plants expressing *At4g32460* antisense RNA, PME activity was decreased in the leaves, and the siliques were very short and contained no seeds. This phenotype was also present in the SALK_142260 and SALK_054867 lines for *At4g32460*.

**Conclusions:**

Our results suggested that the DUF642 family contributes to the complexity of the methylesterification process by participating in the fine regulation of pectin status during plant development.

**Electronic supplementary material:**

The online version of this article (doi:10.1186/s12870-014-0338-8) contains supplementary material, which is available to authorized users.

## Background

DUF642 proteins constitute a highly conserved family of cell wall-associated proteins specific to spermatophytes [1]. Although proteins in this family have been detected in cell-wall proteomes from a variety of plants and tissues, only one functional study on this protein family has been published so far. *At5g25460* is highly expressed in seedlings during the early developmental stages, and plants of the *At5g25460*-null mutant have shorter roots and smaller rosettes than those of wild-type plants [2].

Transcriptome analyses have revealed the differential expression of five DUF642 genes during seed barley germination, suggesting a possible function of this protein family during the germination process [3]. In addition, differential spatial expression of DUF642 genes among various seed compartments during germination has been reported in *Arabidopsis thaliana*. A gene expression study showed that *At3g08030* and *At5g11420* transcripts are enriched in the micropylar endosperm before testa rupture, whereas *At4g32460* is expressed in this compartment after testa rupture [4]. In *Brassica oleracea* seeds, the expression of the *At5g25460* gene ortholog increases during germination [5]. *At3g08030* transcript is present in after-ripened seeds, and the transcript levels increased in seeds subjected to controlled imbibitions in soil or water (matrix-primed and hydroprimed seeds). Notably, *At3g08030* transcript is absent from aged seeds with low germination performance [6].

DUF642 proteins have been detected in the cell-wall proteomes of multiple tissues [7]. A transcriptome analysis of stigmatic papillae cells revealed high transcript levels of two DUF642 genes, *At4g32460* and *At2g41800*, and genes encoding other cell wall-related proteins, including a pectin methyl esterase. Cell wall remodeling of the stigma is involved in successful pollination, likely via regulating the penetration of the pollen tube through the transmitting tract [8]. In *Lilium longiflorum*, an analysis of proteins in the stigmatic exudate revealed a DUF642 protein [9].

Proteins encoded by *At4g32460* and *At5g11420* interact in vitro with the catalytic domain of pectin methyl esterase 3 (AtPME3, encoded by *At3g14310*) [10]. Protein interactome data have proved to be an useful resource for formulating and testing hypotheses [11]. One potential physiological function of the DUF642 proteins encoded by *At4g32460* and *At5g11420* is related to the regulation of PME activity. Several studies have shown that the degree of pectin methylesterification, a highly regulated process, is critical for fine-tuning the biomechanical properties of the cell wall during various developmental processes [12–14]. The demethylesterification of pectins is mediated by PMEs, and PME catalytic activity is regulated by PME inhibitor (PMEI) proteins [15].

Unesterified pectins, especially homogalacturonans (HGs), are the substrates for polygalacturonases (PGs), enzymes regulated by polygalacturonase inhibitor proteins (PGIPs) that are involved in cell separation processes [16]. Differences in pectin methylesterification have been described during pistil, silique, and seed development. In olive, low methylesterified HGs are detected in the stigma and in the transmitting tissue during pollination [17]. In *A. thaliana*, silique growth is related to a decrease in the degree of methylesterification [18]. In seeds of *A. thaliana*, the cell walls within the embryo have low levels of unesterified pectins, the endosperm cell walls contain abundant unesterified HG, and the testa cell walls are rich in highly methylesterified HG [19]. In *A. thaliana,* the genes encoding pectin-modifying enzymes and their regulators are highly regulated during the first 24 h of seed germination [20]. In yellow cedar seeds, PME activity positively correlates with germination performance [21].

In *A. thaliana* and related endospermic species, germination is a two-step process that requires testa and endosperm rupture for radicle protrusion [22]. During *A. thaliana* germination, PME activity increases until testa rupture is complete, and decreases during endosperm breakdown. Overexpression of PMEI led to accelerate endosperm breakdown and an improved capacity for radicle emergence. Delays in endosperm rupture caused by abscisic acid significantly extend the period of high PME activity [13]. Conversely, PGIP overexpression inhibits germination, a process that is enhanced in *pgip* mutant seeds [23].

The aim of this study was to study the function of the DUF642 gene *At4g32460* during seed germination and plant development. We evaluated the role of the BDX protein in the regulation of PME activity, focusing on the periods of seed germination and plant growth. We demonstrated that the overexpression of either *At4g32460* or its homolog *At5g11420* increased PME activity and promoted germination, primarily by accelerating testa rupture. We also demonstrated that total PME activity was inhibited in *At4g32460* antisense transgenic plants and that the morphological changes in these plants included small siliques with no seeds. This phenotype was also observed in SALK T-DNA mutants. In accordance with these results, we named *At4g32460* as *BIIDXI* (*BDX*), which means ‘seed’ in the Zapotec language. Our data suggest that DUF642 proteins are involved in the regulation of PME, thereby remodeling the cell wall during various processes in plant development.

## Results

**Figure 1:**
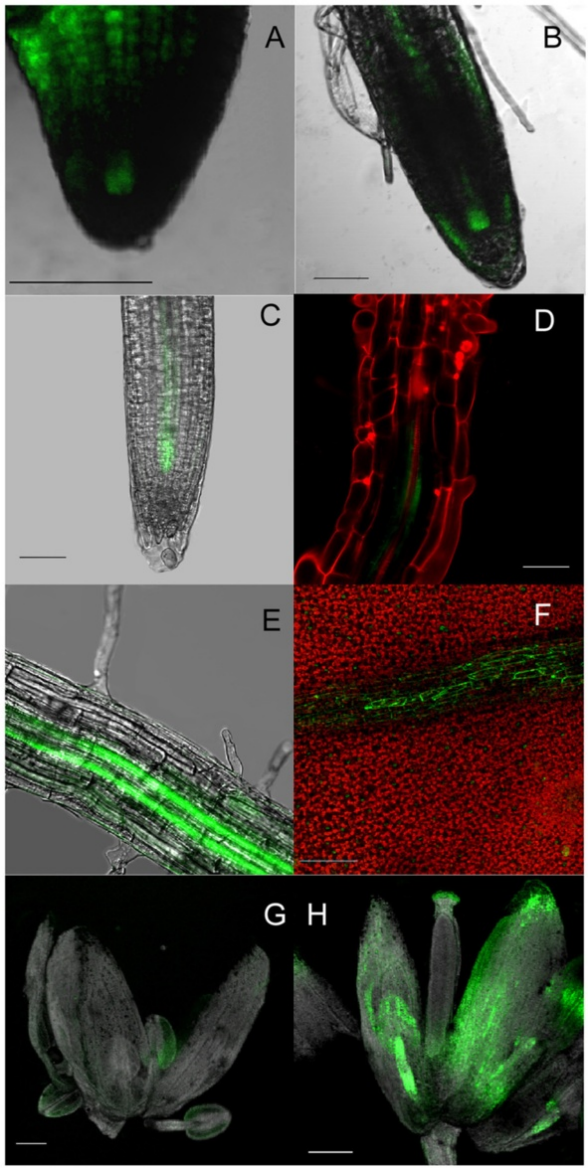
**Identification of** ***At4g32460*** **promoter activity during** ***Arabidopsis thaliana*** **development using pBDX::ER-GFP plants (Additional file** 1 **: Figure S1C). A)** GFP fluorescence in radicle tissue of a 48-h germinating seed. **B)** GFP fluorescence in radicle tissue of a 72-h germinating seed. **C)** GFP fluorescence in different cell types of meristematic and transition zones of primary roots from 4-day-old seedlings. **D)** GFP fluorescence in vascular tissue of maturation zone of primary roots of 22-day-old plants. **E)** GFP fluorescence in pericycle-differentiated cells that constitute primordium of lateral root emerging from maturation zone of primary roots of 22-day-old plants. **F)** GFP fluorescence in vascular tissue of fully expanded leaves. **G)** GFP fluorescence in anthers and petals of stage-6 flowers. **H)** GFP fluorescence in vascular tissue of stamen filaments, anthers, petals, and stigma of stage-12 flowers. Scale bars =50 μm in A and B, 15 μm in D, 100 μm in E and F, and 300 μm in **C**, **G**, **H**, and **I**. Images **A**, **B**, **E**, **F**, **G**, and **H** are projections of confocal Z-stacks. C and D are longitudinal sections.

*BIIDXI* is expressed in the embryos of imbibed seeds, roots, leaves, stems, and various floral organs (bar.utoronto.ca). To determine whether the cloned region (Additional file 1: Figure S1C) was sufficient to drive expression in a pattern similar to that described previously for *A. thaliana*, we produced transgenic plant lines containing the cloned fragment fused to the green fluorescent protein (GFP) reporter. Three transgenic lines were produced, and we monitored GFP fluorescence throughout their growth and development. GFP driven by the *BDX* promoter was highly expressed in the vascular tissue of primary and lateral roots, and in leaves, stamens, and petals (Figure 1). GFP fluorescence was detected in the vascular tissue of radicles from seeds that had been germinating for 48 h and 72 h (Figure 1A and B). During the seed imbibition process, GFP fluorescence was detected from 6 h until germination was complete (Additional file 1: Figure S2). In the primary roots of 6-day-old seedlings, GFP fluorescence was detected exclusively in the provascular tissue of the meristematic and transition zone (Figure 1C). In the roots of 8-day-old seedlings, GFP fluorescence was detected in pericycle cells in the differentiation zone (Figure 1D). In the roots of 22-day-old plants, GFP fluorescence was detected in the vascular tissue in specific regions of the mature zone (Figure 1E). GFP fluorescence was also detected in the vascular tissue of fully expanded leaves (Figure 1F). During different stages of flower development, *BDX* promoter-driven GFP expression was detected exclusively in the vascular tissue of stamen filaments and anthers, in petals, and in the stigmatic papilla, as described previously [8] (Figure 1G and H).

**Figure 2:**
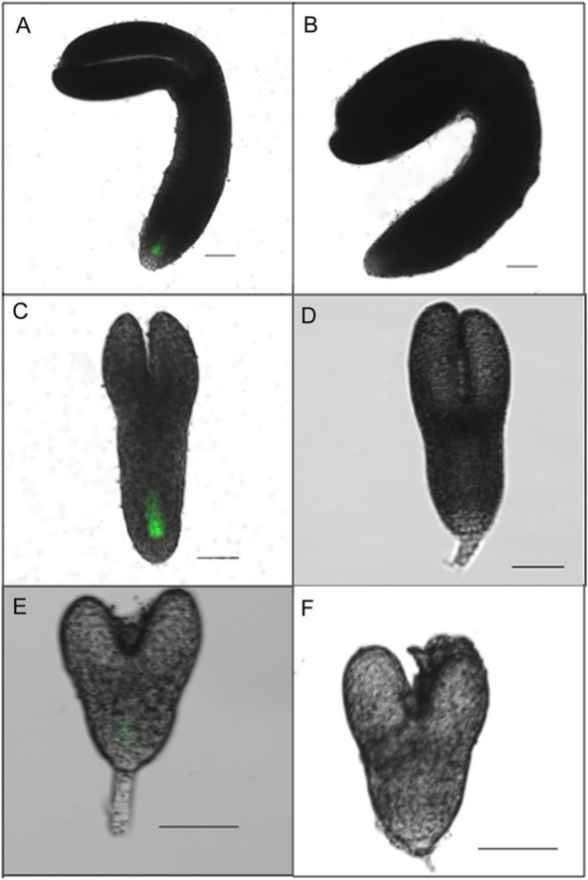
**Identification of** ***At4g32460*** **promoter activity during** ***Arabidopsis thaliana*** **embryo development using pBDX::ER-GFP plants (Additional file** 1 **: Figure S1C). A)** GFP fluorescence in provascular cells from radicle meristematic region of mature embryo. **C)** GFP fluorescence in provascular cells from radicle meristematic region of torpedo-stage embryo. **E)** GFP fluorescence in provascular cells from radicle meristematic region of heart stage embryo. **B)**, **D)**, and **F)** Images of wt embryo stages. Scale bars =50 μm. All images are projections of confocal Z-stacks.

Next, we analyzed *BDX* promoter activity in embryos at various stages; heart stage, torpedo stage, and mature embryos (Figure 2). GFP fluorescence was detected in provascular cells from the radicle meristematic region of the mature embryo (Figure 2A) and in embryos at the torpedo (Figure 2C) and heart stages (Figure 2E). The expression pattern of GFP driven by the *BDX* promoter was primarily associated with vascular tissue during different stages of plant development, consistent with previous reports.

Transcriptome analyses have revealed that *BDX* expression is induced by auxin [24] and also by gibberellic acid (GA) during germination [25]. In silico analysis of the putative promoter region (pBDX) revealed at least two auxin response factor motifs [26] and two gibberellic acid response element (GARE) [27]. We performed hormone induction analyses to test whether the pBDX fragment contained information for auxin and GA responses. Auxin and GA treatments, for 2 h or 48 h, altered *BDX* expression in the roots of 7-day-old seedlings. In both treatments, *BDX* expression was detected in vascular tissue, as previously observed in control seedlings, but also in cortical cells (Additional file 1: Figure S3).

**Figure 3:**
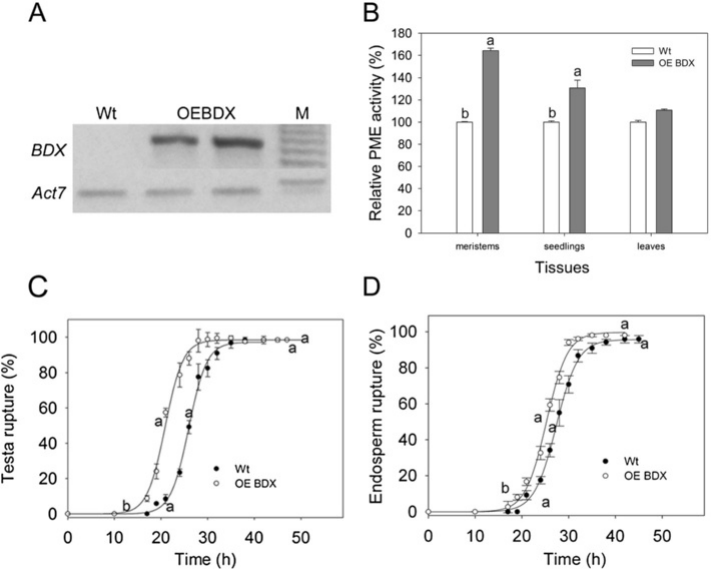
**Effects of** ***BDX*** **overexpression on** ***BDX*** **transcript level, PME activity, and germination of matrix-primed seeds. A)** Detection of *BDX* transcripts in dry seeds from wt plants and two independent OEBDX lines. *ACT7* expression was used as an internal control. **B)** PME activities in meristem and leaves of *A. thaliana* plants (18 days after sowing) and seedlings (5 days post-germination). **C)** Cumulative testa rupture curve. **D)** Cumulative endosperm rupture curve. Germination assays were performed in triplicate at 20°C. All experiments were performed at least three times for each line, with different seed lots. Values shown are mean ± standard error; different letters indicate significant differences among lines.

To understand the physiological function of *BDX*, we generated overexpression lines (OEBDX; Figure 3) in which the full-length *At4g32460* coding sequence was expressed under the control of the cauliflower mosaic virus 35S promoter (Additional file 1: Figure S1A). Several independent and homozygous transgenic lines were obtained. We examined dry seeds from two lines to determine their PME activity and *BDX* transcript levels.

*BDX* transcript was not detected in wt dry seeds, but was presented at high levels in the overexpression lines (Figure 3A). Although there were no morphological differences among the different lines at all developmental stages (results not shown), there was increased total PME activity in vegetative-meristem-enriched samples and in seedlings (*p* <0.001), compared with that in wt. There was no significant difference in PME activity in leaves between the overexpression lines and wt (Figure 3B). In wt *A. thaliana* seeds, PME activity has been reported to increase before testa rupture and to decrease at the beginning of endosperm rupture [13]. Based on this information, we performed a germination analysis of matrix-primed seeds of two OEBDX lines and wt plants. Plants transformed with the empty vector were used as a negative control (Additional file 1: Figure S4). Compared with wt, OEBDX lines showed a shorter initial time of testa rupture (*p* =0.02, Figure 3C), and their seeds showed a shorter time to endosperm break initial time (*p* =0.004, Figure 3D).

**Figure 4:**
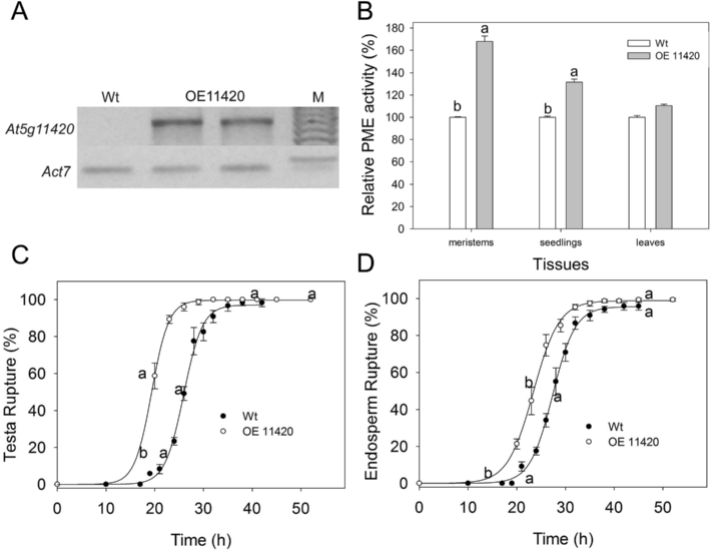
**Effects of** ***At5g11420*** **overexpression on** ***At5g11420*** **transcript level, PME activity, and germination of matrix-primed seeds. A)** *At5g11420* transcript level in dry seeds of wt and two independent OE11420 lines. *ACT7* expression served as the internal control. **B)** PME activities in meristem and leaves of *A. thaliana* plants (18 days after sowing) and seedlings (5 days post-germination). Values are means ± standard error. **C)** Cumulative testa rupture curve. **D)** Cumulative endosperm rupture curve. Germination assays were performed in triplicate at 20°C. Different letters indicate significant differences among lines. All experiments were performed at least three times for each line, with different seed lots.

Phylogenetic studies have demonstrated that *BDX* and *At5g11420* resulted from a recent duplication event, and that their respective encoded proteins interact with AtPME3 in vitro [1,10]. Also, recent studies have shown that *At5g11420* expression in the micropylar endosperm increases prior to testa rupture, suggesting a potential role for *At5g11420* in this process [4]. To evaluate the possible role of *At5g11420* in testa rupture, we generated overexpression lines (OE11420, Figures 1B and 4). There were no morphological differences among the different lines and wt (results not shown). *At5g11420* transcript was detected at high levels in dry OE11420 seeds (Figure 4A). Compared with wt, the OE11420 lines showed a significant increase in PME activity in vegetative-meristem-enriched samples and seedlings (*p* =0.003), but no significant change in PME activity in the leaves (Figure 4B). Also, OE11420 matrix-primed seeds showed improved germination, compared with that of wt seeds (Figure 4C and D). Specifically, the lag time until the initiation of testa and endosperm rupture was shorter in OE11420 seeds than in wt seeds (*p* =0.004 and *p* < 0.001, respectively). There was an increase in the rate of endosperm rupture in OE11420 lines (*p* <0.001). Germination analysis of OEBDX and OE11420 matrix-primed seeds suggests that the overexpression of these genes in dry seeds improves their germination, possibly through enhancing testa rupture performance (Figures 3 and 4).

**Figure 5:**
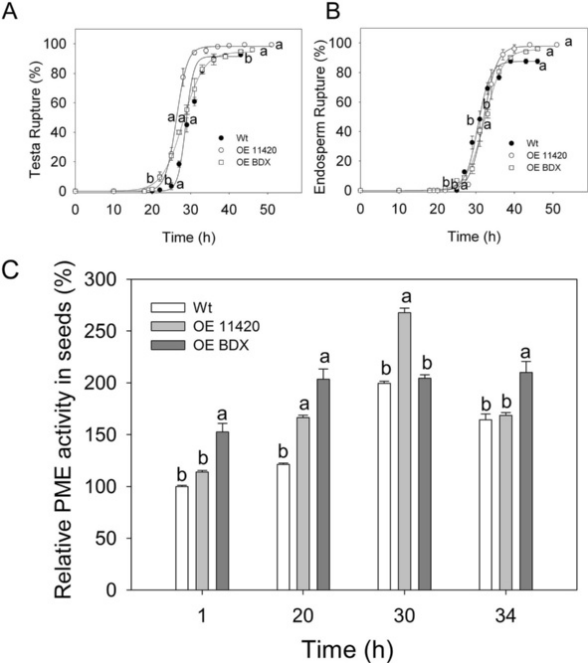
**PME activity during germination of OEBDX and OE11420 control (dry) seeds. A)** Cumulative testa rupture curve. **B)** Cumulative endosperm rupture curve. **C)** PME activity. PME activity assays were performed in triplicate. All experiments were performed at least twice for each line, with different seed lots. For each time point, letters indicate significant differences. Error bars show SE.

To determine the effects of the overexpressed genes on seed germination and PME activity during germination, we tested seeds without a priming pre-treatment. The initial testa rupture and endosperm rupture times were shorter for OE seeds than for wt seeds (*p* =0.01 and *p* < 0.01, respectively). The seeds of OEBDX and OE11420 did not show different testa rupture rates, but seeds of both lines showed significantly lower endosperm rupture rates compared with that of wt seeds (*p* =0.01, Figure 5A and B). For wt seeds, PME activity increased before testa rupture was complete and decreased thereafter, similar to the pattern reported previously [13]. The same pattern of PME activity was observed in OE11420 seeds. At 20 h of germination, PME activity was significantly higher in OE11420 seeds than in wt seeds. However, the PME activity pattern for OEBDX seeds was different; the PME activity did not decrease at 34 h of germination. In the 1-h and 34-h germinating seeds, PME activity was higher in OEBDX seeds than in wt seeds (*p* <0.001, Figure 5C).

Pectins are the main component of *A. thaliana* seed mucilage, and can be detected by staining with ruthenium red [28]. In water-imbibed OEBDX seeds, mucilage release was similar to that of wt imbibed seeds (Additional file 1: Figure S5).

**Figure 6:**
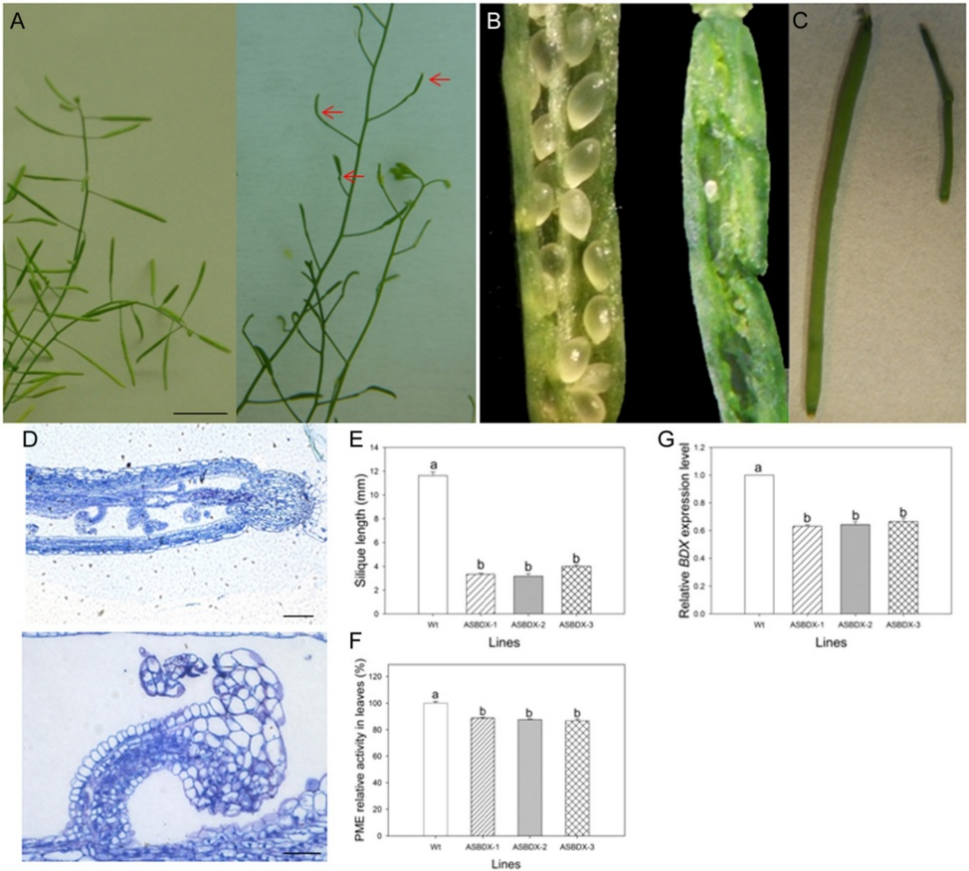
**Phenotypic, molecular, and biochemical analyses of pBDX::BDX antisense transgenic lines. A)** Representative wt and ASBDX plants, arrows indicate differences in siliques respect to wt. **B)** Representative open siliques from wt and ASBDX plants **C)** Close up from wt and ASBDX siliques. **D)** Longitudinal section of pistil from stage 13 flower from ASBDX. Scale bar =200 μm. A close-up of one ovule is shown. Scale bar =25 μm. **E)** Quantitative analysis of silique length in wt and ASBDX lines 1, 2, and 3. **F)** PME activity in total protein extracts from rosette leaves of wt and ASBDX lines 1, 2 and 3. For all lines, experiments were performed at least three times with different plants. Values shown are mean ± standard error. **G)** Expression of pBDX::BDX antisense transgene significantly decreased endogenous *BDX* transcript level in inflorescences of all three lines. Different letters indicate significant differences.

Next, we used antisense RNA technology to silence *BDX* expression. An *At4g32460* RNA antisense transgene driven by the cognate promoter of the endogenous *BDX* gene (BDX::BDX antisense RNA transgene; Additional file 1: Figure S1D) was transformed into wild-type *A. thaliana* plants to generate ASBDX plants (Figure 6). Five independent transgenic lines were obtained by kanamycin selection. All of these transgenic lines exhibited phenotypic variation in the T1 progeny, and the segregation analysis revealed a 3:1 ratio of wt phenotypes to defective phenotypes. This ratio was previously observed for an antisense construct with a cognate promoter in rice [29]. Analyses of morphological and developmental defects were conducted using three independent antisense lines. All three lines showed reduced silique size and did not produce seeds (Figure 6A, B, C and D). Analysis of a transverse section of a stage 13 ASBDX flower bud revealed abnormal carpel morphology. The size of the stigma was decreased, and the septum had engrossed regions. In mature ovules, different tissues could not be differentiated (Figure 6D, close-up image). The mean silique length for ASBDX lines 1, 2, and 3, was 3.3, 3.1, and 4 mm, respectively, corresponding to a size reduction of at least 60% (Figure 6E). Compared with wt, all three ASBDX lines showed significantly lower PME activity in the leaves (*p* =0.014, *p* =0.001, and *p* =0.003 for lines 1, 2, and 3, respectively; Figure 6F). A quantitative reverse-transcription polymerase chain reaction (qRT-PCR) analysis revealed that all three ASBDX lines showed a 40% reduction in *At4g32460* transcript levels, compared with that in wt inflorescences (*p* <0.001 for line 1 and 2, and *p* =0.004 for line 3; Figure 6G).

**Figure 7:**
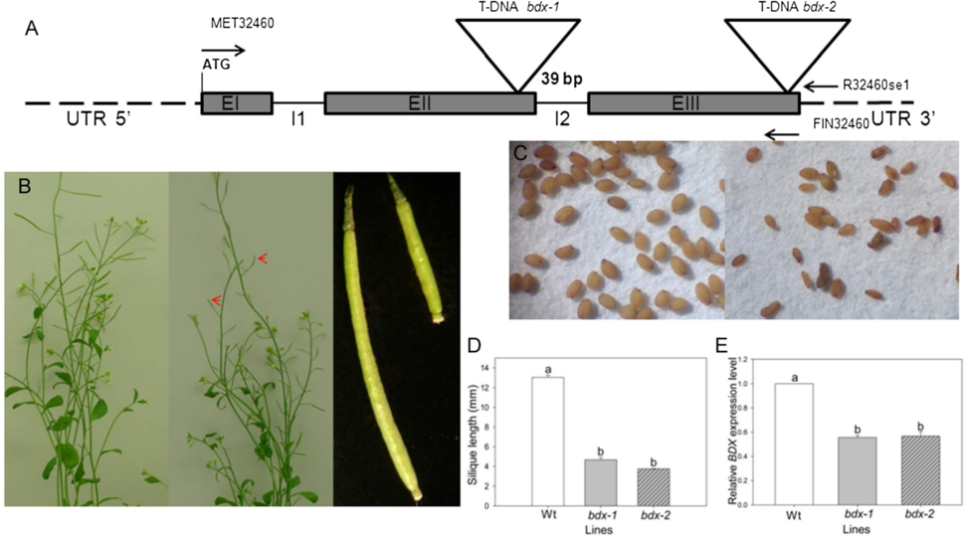
**Phenotypic, molecular, and biochemical analyses of T-DNA insertion lines** ***bdx-1*** **and** ***bdx-2*** **. A)** Schematic representation of T-DNA insertion at end of second exon of *BDX* for *bdx-1* and at end of third exon for *bdx-2*. **B)** Phenotypic comparison between heterozygous *bdx-1* plant and wt. All siliques from heterozygous *bdx-1* plants were shorter than those from wt. **C)** Many seeds from *bdx-1* plants were small and wrinkled (right). Similar phenotype was observed for T-DNA *bdx-2* siliques and seeds (Additional file 1: Figure S6). **D)** Quantitative analysis of silique length in wt, *bdx-1*, and *bdx-2*. **E)** *BDX* endogenous gene expression was significantly decreased in inflorescences of heterozygous *bdx-1*and *bdx-2,* compared with that in wt inflorescences. Different letters indicate significant differences.

We conducted a phenotypic analysis of two SALK lines, SALK_142260 and SALK_054867, each of which have a T-DNA insertion in the *BDX* coding sequence. The results provided further evidence that *BDX* plays roles in reproductive development, and possibly in modulating PME (Figure 7). The T-DNA insertion in the SALK_142260 line is at end of the second exon and that in the SALK_054867 line at the end of the third exon of the *At4g32460* locus (Figure 7A). Heterozygous plants of the two SALK lines showed a reduction in silique length, similar to that in ASBDX plants (Figure 7B and D). However, in the SALK lines, the siliques contained a few seeds (average, 4 ± 1 seeds). Some seeds showed an abnormal morphology and were not viable (Figure 7C). Seeds with normal morphology generated either heterozygous or wt plants but not homozygous plants. A qRT-PCR analysis showed a significant decrease in *BDX* transcript levels in T-DNA heterozygous plants (*p* =003, Figure 7E).

## Discussion

DUF642 is a highly conserved family of cell wall-related proteins specific to spermatophytes. This family shows a high level of amino acid identity among different plant species, suggesting that members of the DUF642 play an important function in plant cell wall properties [1]. Our results showed that *BDX* was expressed in embryos, in imbibed seeds, in 48-h seedlings, and throughout the adult plant, where it was primarily localized in vascular tissue, as has been described for *At5g25460* [2] (Figures 1 and 2). *BDX* was detected in the initial cells of the different stages of the embryo and primary root, mainly in the pericycle cells before lateral root emergence; therefore, its expression in vascular tissue in other organs could be related to these types of cells.

Auxin signaling promotes the emergence of lateral root primordia [30]. In the present study, auxin induced *BDX* expression in roots (Additional file 1: Figure S2), and *BDX* was highly expressed in stigma tissue of stage 12 flowers (Figure 1H), where auxin-signaling genes are overrepresented [31]. Auxin distribution in the stigma plays an important role in the pollination process. In the ASBDX plants, the siliques were very short and contained no seeds; in the heterozygous T-DNA plants, the siliques were short but they contained a few seeds (Figures 6 and 7). The high transcript level of *BDX* in stigma tissue could be related to the low seed yield of ASBDX and T-DNA plants. It has been suggested that the regulation of cell wall structure, and especially pectin status, in female tissue is fundamental for pollen tube penetration [32]. Previous studies have shown that methylesterification of the transmitting tract decreases before and during pollination and that PME activity increases during silique development [17,18,32]. Whereas *pme3*-null mutants showed no morphological changes in silique development [33], PMEI5 OE plants grew short and wrinkled siliques containing only one or two seeds. A decrease in total PME activity is detected in PMEI5 OE [13] and in ASBDX plants (Figure 6).

BDX was also expressed during embryogenesis (Figure 2). In three generations, homozygous T-DNA plants were not obtained, suggesting that there was an embryo development defect in null mutant plants.

Transgenic plants overexpressing AtPME3 showed an increase in total PME activity, but their only morphological changes are longer roots and taller shoots than those of wt [33]. In the present study, the *A. thaliana* plants overexpressing *BDX* and *At5g11420* had no morphological changes, as compared with wt, during different vegetative stages of the life cycle, although they showed higher total PME activity in the meristem, seedlings, and imbibed seeds (Figures 3 and 4). The increased PME activity could be related to improved germination performance in OEBDX and OE11420 seeds. Compared with wt seeds, OEBDX and OE11420 seeds showed shorter initial times to testa rupture, which were correlated with the increase in PME activity (Figure 5). A previous study showed that the *At5g11420* transcript is enriched in the micropylar endosperm before testa rupture [4]. In the present study, there were high levels of PME activity in the hours before testa rupture in the OE11420 line, suggesting that *At5g11420* plays a physiological role in germination.

## Conclusions

Our data indicated that the DUF642 proteins encoded by *BDX* and *At5g11420* are positive regulators of PME activity in *A. thaliana*. They may affect PME activity directly, or indirectly via altering cell wall properties. The relevance of cell wall modifications during plant development has been studied extensively, and PMEs have been shown to play important roles in modulating methylesterification during fruit development and germination [34]. In this study, the lines overexpressing *BDX* and *At5g11420* showed increased PME activity during seed germination, a two-step process that is highly correlated with changes in PME activity. We speculate that PME activation leads to faster completion of germination by improving the capacity for testa rupture. Previous studies have shown that reduced cell wall pectin methylesterification allows improved access of PG to degrade pectin and promote cell separation in the testa. In addition, the modulation of PG activity by PGIP was shown to inhibit germination [23].

Many morphogenetic events during algal and plant development are related to pectin chemistry [34,35]. In *Chara corallina*, pectin de-esterification promotes cell expansion [36]. Phyllotaxis and organ initiation depend on the regulation of pectin status of the meristem [37]. During pollen tube elongation, a spatial gradient in pectin methyl-esterification is shown to be precisely controlled by PMEs and PMEI proteins [38].

The evolution of the complexity of the methylesterification process is an important process in plant diversification and adaptation to different environments. The expression of pectin and PME in cell walls first occurred in charophytes. The inhibitory domain of PMEs appeared in the PMEI family in land plants during the divergence of mosses from charophytes [39]. The cellulose binding protein (CBP) of the nematode *Heterodera schachtii* increases PME activity in plants through directly interacting with AtPME3; other than this exception, no other positive regulator of PMEs has been described [33]. Our results suggest that DUF642 proteins, a spermatophyte-specific family, contribute to the complexity of the methylesterification process by participating in the fine regulation of pectin status during plant development.

## Methods

### Plant materials

Wild-type Columbia (Col) ecotype *A. thaliana* plants (wt) and lines overexpressing (OE) *At4g32460* and *At5g1120* were sown in soil in pots, or on Murashige and Skoog (MS) medium (pH 5.7) in Petri dishes. The plants were grown in a chamber (E-15, Conviron, Manitoba, Canada) at 21°C under a long-day photoperiod (16-h light/8-h dark). Five-day-old seedlings and vegetative meristems (comprising the young organ primordia and the shoot apical meristem proper) and rosette leaves (from 18-day-old plants) were collected, frozen in liquid nitrogen, and stored at −80°C until use in PME activity and RNA analyses.

To determine the expression patterns of *At4g32460* and *At5g11420* during imbibition, 50 mg wt *A. thaliana* seeds were sown on 1% (w/v) agar plates and placed in a growth chamber (Lab-Line Instruments Inc. Melrose Park, IL, USA) at 21°C under a 16-h light/8-h dark photoperiod. Seeds were collected at 2, 4, 6, 8, 12, 24, and 48 h post-imbibition, frozen in liquid nitrogen, and then stored at −80°C until RNA extraction.

### DNA extraction, RNA extraction, and seed cDNA synthesis

Genomic DNA was extracted from rosette leaves and inflorescences using the phenol chloroform isoamyl alcohol method (Invitrogen, Carlsbad, CA, USA) according to the manufacturer’s instructions. Total RNA was extracted from rosette leaves and inflorescences using the Trizol method (Invitrogen) according to the manufacturer’s instructions.

Total RNA was isolated from 0.05 g *A. thaliana* wt and OE seeds according to the protocol described elsewhere [40]. A Nanodrop spectrophotometer (Nanodrop Lite; Thermo Scientific; www.thermoscientific.com) was used to quantify RNA. For semiquantitative RT-PCR, cDNA was synthesized from 100 ng RNA that had been treated with DNase-I (Qiagen, Valencia, CA, USA) using SuperScript II Reverse Transcriptase (Invitrogen) and oligo (dT) primers. We used the primer pair FAT32460 (forward, 5′-GTGTCCCAAAGCCATTATTC-3′) and RAT32460 (reverse, 5′-AGCGACGAATCTCAATGAC-3′) to amplify *At4g32460*, and the primer pair 11420LSF (forward, 5′-TCTAGAATGAAAGGAGGCAGCCTCT-3′) and 11420LSR2 (reverse, 5′-GGATCCCGGCTTACGAGCACTGAGGAGTT-3′) to amplify *At5g11420. Actin* (*ACT7*) was used as an internal control.

### Quantitative RT-PCR

cDNA samples synthesized from 100 ng RNA from antisense, T-DNA, and wt inflorescences were used for amplification with SYBR Green Master Mix using an Applied Biosystems StepOne platform (Applied Biosystems, Foster City, CA, USA). The PCR conditions were as follows: 50°C for 2 min for DNA polymerase activation, 95°C for 10 min, followed by 40 cycles of 15 s at 95°C and 1 min at 60°C; finally, samples were subjected to 15 s at 95°C, 1 min at 60°C, and 15 s at 95°C for melting curve analysis. Three independent biological replicates with three technical replicates were analyzed. *ACT7* served as the endogenous control. To analyze gene transcript levels in inflorescences of antisense plants, the forward primer consisted of a sequence from the UTR 5′ region (5′-CTCTCGCTCACTCTTCTCCAA-3′) and the reverse primer consisted of a sequence from the beginning of the second exon (5′-CGACAAAGCCTGAGAGTTCCC-3′, BDXR). For analyses of the T-DNA lines, we used the *BDX* forward primer (5′-GCTTCAATGATGGACTACTACC-3′, BDXF) and BDXR. Samples were compared with C_T_ and slope values and analyzed using the mathematical model established by Pfaffl [41] to obtain the relative expression ratio. Data were subjected to a natural log transformation before the Student’s *t*-test.

### Plasmid construction

For *At4g32460* (GENE ID 829381) expression analysis, a 1983-bp fragment of the *At4g32460* intergenic region ending at ATG was amplified from leaf-extracted genomic DNA using the following primers: PRO32460F (forward, 5′-AAGCTTGCATGGGAGAATTGACCACT-3′) and PRO32460R (reverse, 5′-GGATCCTTGGAGAAGAGTGAGCGAGAG-3′). This PCR fragment was cloned into pGEM-T Easy and then sequenced. The fragment was then partially digested with HindIII followed by BamHI, and then cloned into the pBIN-m-GFP-ER plasmid (PRO32460: ER-GFP, Figure 1).

For overexpression of *At4g32460* and *At5g11420* (GENE ID 831013), their coding regions were amplified from cDNA synthesized from leaf mRNA using the primers pairs F32460se1 (forward, 5′-GGATCCATGAAAGAGATGGGAGTGATAG-3′) and R32460se1 (reverse, 5′-GAGCTCTCACGGCCTCCGAGCACT-3′); and F11420se1 (forward, 5′-GGATCCATGAAAGGAGGCAGCCTCT-3′) and R11420se1 (reverse, 5′-GAGCTCTTACGGCTTACGAGCACTGA-3′), respectively. These PCR fragments were cloned into pGEM-T Easy and then sequenced. After digestion with BamHI and SacI, the fragments were subcloned into the pBIN plasmid and expressed under the control of the CaMV 35S promoter (Figure 1C and D). For gene silencing analysis, we constructed a plasmid with the *At4g32460* antisense transgene under the control of its cognate promoter.

### Plant transformation

Wt Col *A. thaliana* plants were transformed using the floral-dip method via *Agrobacterium tumefaciens* C58 [42]. Collected seeds were sown on plates containing MS medium (pH 5.7) with kanamycin for selection, and green seedlings were transplanted into soil in pots. At least 10 independent transgenic lines were selected. The homozygous T3 seed generation was used for germination experiments (two independent lines) or confocal microscopy (three independent lines). For antisense transgenic plants, at least 10 independent lines were selected. For phenotype analysis, five homozygous (T3) lines were used, and three lines were used for PME activity and qRT-PCR analyses.

### T-DNA lines

The insertion in the T-DNA SALK_142260 line was verified by PCR, and the region of interest was sequenced using the T-DNA left border primer LBb1 (5′-GCGTGGACCGCTTGCTGCAACT-3′) and a specific primer for *BDX,* R32460se1, located at the end of the second exon*.* For the SALK_054867 line, the reverse primer consisted of a sequence located at the end of the third exon.

### Laser confocal scanning microscopy

To label the cell walls, seedlings were incubated in a propidium iodide solution (1.7 mM) for 30 s before confocal imaging analysis. Propidium iodide and GFP were imaged at 485 and 545 nm, respectively, using an aFV100 Laser Confocal Scanning Microscope (Olympus, Tokyo, Japan). Images from all plant tissues without fluorescence were used as controls. The images were assembled using Photoshop, v. 5.0 (Adobe Systems, San Jose, CA, USA).

### Matrix priming treatment (M)

*A. thaliana* seeds were enclosed in cellophane and buried at 2 cm depth in a pot filled with field-capacity humid soil (−0.027 MPa water potential). The pot was covered with aluminum foil and incubated at 22°C for 24 h. Seeds were exhumed in the dark and air-dried at room temperature (25°C). Subsequently, the seeds were used for germination testing or frozen in liquid nitrogen and stored at −80°C.

### Germination testing

For germination testing, dry mature seeds were stored at 20°C for 3 months of after-ripening. Seeds were sown on 1% (w/v) agar plates and transferred to a plant growth chamber (Lab-Line Instruments Inc.) at 20°C under a 6-h light/18-h dark photoperiod. All germination experiments consisted of five replicates with 30 seeds per replicate. Biological replicates were performed with different seed lots of wt and the different overexpression lines for *BDX* and *At5g11420*. Germination was scored using an Olympus dissection microscope. Seeds with visible endosperm were considered to have reached testa rupture. Seeds with a radicle tip emerging through the endosperm were considered to have reached endosperm rupture. The percentages of seeds with testa and endosperm rupture over time were fitted to sigmoid models using Table Curve 2D v.3 software (AISN, Software, Chicago, IL, USA). From these models, we obtained the lag time of germination and the germination rate after an arcsine transformation to meet the assumptions of the test. The results were analyzed with an analysis of variance (ANOVA) and Tukey’s post hoc test (Sigma Plot v.11, Systat Software Inc., San José, CA, USA). Germination percentages were also arcsine-transformed to meet the assumptions of the test. To compare the slope between wt and OE lines, the testa and endosperm fits were included.

### PME activity assays

PME activity was determined according to a previously described method [28] with the following modifications: 8–20 μg protein in equal volumes (5–20 μl) was loaded into a gel matrix prepared in 50-mm Petri dishes (instead of 6-mm-diameter wells). The gel consisted of 0.1% (w/v) ≥85% esterified citrus fruit pectin (Sigma-Aldrich), 1% (w/v) agarose, 12.5 mM citric acid, and 50 mM Na_2_HPO_4_ (pH 6.5). The PME activities in different tissues were normalized to the corresponding wild-type average activity (=100). Significant differences were assessed using a Mann–Whitney *U* test and two-way multivariate analysis of variance (MANOVA) for seed imbibition.

### Histological analyses

Silique samples from plants harboring the antisense construct were fixed in FAA (by volume, 4% formaldehyde, 2% acetic acid, 50% ethanol). The samples were dehydrated through a graded ethanol series and embedded in paraffin. Sections (1–2 μm) were cut with a rotary microtome, and then stained with toluidine blue. Histological sections were observed and photographed under an Olympus microscope.

### Availability of supporting data

The supporting data of this article is included in the additional file.

## Additional file

Additional file 1: Figure S1. Schematic presentation of transgene constructs. (**A**, **B**) *At4g32460* and *At5g11420* cDNA fragments were cloned into pBIN to generate CAMV 35S::BDX (OEBDX) and CAMV 35S::*At5g11420* (OE11420) constructs, respectively. **(C)** To construct pBDX::mGFP-ER, a 1983-bp fragment of *At4g32460* intergenic region was cloned into pBIN-m-GFP-ER. **(D)** pBDX::BDX antisense consisted of *At4g32460* RNA antisense transgene driven by the cognate promoter of *At4g32460* (ASBDX). Black arrow, CaMV promoter; striped arrows, cognate promoters; gray arrows (right), sense fragments; gray arrows (left), antisense fragment. **Figure S2.** Early and differential transcription of *BDX* and *At5g11420* during seed imbibition. *ACT7* was analyzed as internal standard. **Figure S3.** Auxin- and gibberellic acid-inducible expression of *At4g32460* in primary roots*.* Transgenic 5-day-old seedlings were treated for 0 h (**A**, **D**) or 24 h with 2 μM GA (B,E) or for 48 h with 2 μM IAA (**C**,**F**). **A)** GFP fluorescence in provascular tissue of meristematic zone (as in Figure 2). **B)** GFP fluorescence in provascular and vascular tissue of meristematic and transition zone. **C)** GFP fluorescence in provascular and vascular tissue of meristematic and transition zone and was also detected in cortical cells of transition zone. **D)** GFP fluorescence in pericycle cells of vascular tissue of maturation zone (as in Figure 2). **E)** GFP fluorescence in vascular tissue and cortical cells of maturation zone. **F)** GFP fluorescence in vascular tissue and cortical cells of maturation zone. **Figure S4.** Effect of empty plasmid on germination performance. A) Cumulative testa rupture curve. **B)** Cumulative endosperm rupture curve. **Figure S5.** OEBDX and OE11420 seeds did not show defective mucilage release after imbibition in water [43]. **Figure S6.** Phenotypic analyses of *bdx-2*. **A)** Phenotypic comparison between heterozygous *bdx-2* plant and Siliques of heterozygous *bdx-2* plants were shorter than those of wt. **B)** Many seeds from *bdx-2* plants were small and wrinkled (right).

## Abbreviations

*BDX*: *BIIDXI*
DUF642: Domain unknown function 642
PME: Pectin Methyl Esterase
AtPME3: Pectin Methyl Esterase 3 from *Arabidopsis thaliana*
PMEI: PME inhibitor
HGs: Homogalacturanans
PG: Polygalacturonase
PGIP: Polygalacturonase inhibitors proteins
ABA: Abscisic acid
GFP: Green fluorescent protein
GA: Gibberelic acid
GARE: Gibberelic acid response elements
OE: Overexpression
OEBDX: BDX Overexpression
ASBDX: Antisense BDX
CBP: Cellulose Binding Protein
Col: Columbia ecotype
wt: Wild-type
MS: Murashigee and Skoog
IAA: Indole-3-acetic acid
