# Supplementary material for: BIIDXI, the At4g32460 DUF642 gene, is involved in pectin methyl esterase regulation during Arabidopsis thaliana seed germination and plant development
Source: BMC Plant Biol. 2014 Dec 2;14:338. doi: 10.1186/s12870-014-0338-8 (PMC4264326; doi:10.1186/s12870-014-0338-8)
Supplement: Additional file 1: Figure S1. — Schematic presentation of transgene constructs. (A, B) At4g32460 and At5g11420 cDNA fragments were cloned into pBIN to generate CAMV 35S::BDX (OEBDX) and CAMV 35S::At5g11420 (OE11420) constructs, respectively. (C) To construct pBDX::mGFP-ER, a 1983-bp fragment of At4g32460 intergenic region was cloned into pBIN-m-GFP-ER. (D) pBDX::BDX antisense consisted of At4g32460 RNA antisense transgene driven by the cognate promoter of At4g32460 (ASBDX). Black arrow, CaMV promoter; striped arrows, cognate promoters; gray arrows (right), sense fragments; gray arrows (left), antisense fragment. Figure S2. Early and differential transcription of BDX and At5g11420 during seed imbibition. ACT7 was analyzed as internal standard. Figure S3. Auxin- and gibberellic acid-inducible expression of At4g32460 in primary roots. Transgenic 5-day-old seedlings were treated for 0 h (A, D) or 24 h with 2 μM GA (B,E) or for 48 h with 2 μM IAA (C,F). A) GFP fluorescence in provascular tissue of meristematic zone (as in Figure 2). B) GFP fluorescence in provascular and vascular tissue of meristematic and transition zone. C) GFP fluorescence in provascular and vascular tissue of meristematic and transition zone and was also detected in cortical cells of transition zone. D) GFP fluorescence in pericycle cells of vascular tissue of maturation zone (as in Figure 2). E) GFP fluorescence in vascular tissue and cortical cells of maturation zone. F) GFP fluorescence in vascular tissue and cortical cells of maturation zone. Figure S4. Effect of empty plasmid on germination performance. A) Cumulative testa rupture curve. B) Cumulative endosperm rupture curve. Figure S5. OEBDX and OE11420 seeds did not show defective mucilage release after imbibition in water [43]. Figure S6. Phenotypic analyses of bdx-2. A) Phenotypic comparison between heterozygous bdx-2 plant and Siliques of heterozygous bdx-2 plants were shorter than those of wt. B) Many seeds from bdx-2 plants were small and wrinkled (right). [file 12870_2014_338_MOESM1_ESM.pdf]

Additional file

1. **Schematic presentation of the constructed transgenes.**

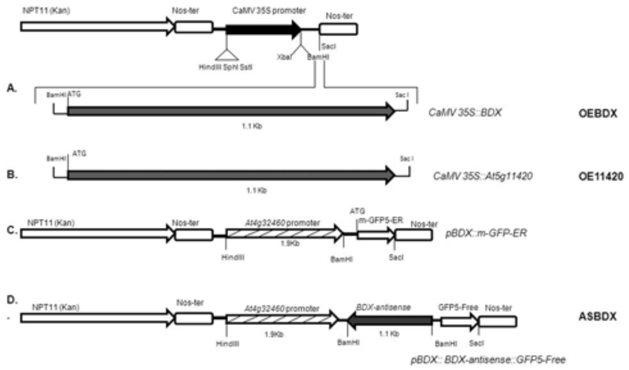

Figure 1. Schematic presentation of transgene constructs. (A, B) Independently amplified *At4g32460* and *At5g11420* cDNA fragments were cloned into pBIN to generate CAMV 35S::BDX (OEBDX) and CAMV 35S::*At5g11420* (OE11420) constructs, respectively. (C) To construct pBDX::mGFP-ER, a 1983-bp fragment of *At4g32460* intergenic region was cloned into pBIN-m-GFP-ER. (D) pBDX::BDX antisense consisted of *At4g32460* RNA antisense transgene driven by the cognate promoter of the endogenous *At4g32460* (ASBDX). Black arrow, CaMV promoter; striped arrows, cognate promoters; gray arrows (right), sense fragments; gray arrows (left), antisense fragment.

2. Early and differential gene expression of *At4g32460* and *At5g11420* during seed imbibition.

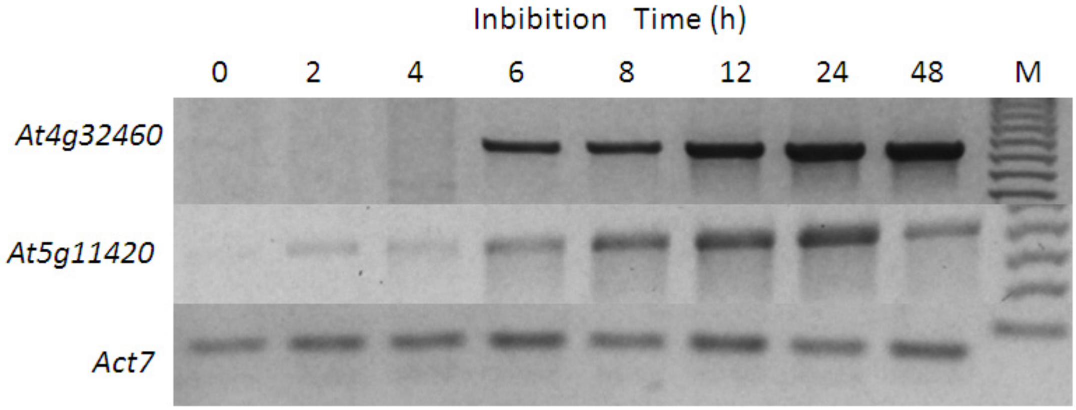

Figure 2. Early and differential transcription of *BDX* and *At5g11420* during seed imbibition. Transcript level of *ACT7* was analyzed simultaneously as an internal standard.

3. Auxin and Giberellic acid promoted *At4g32460* expression in roots.

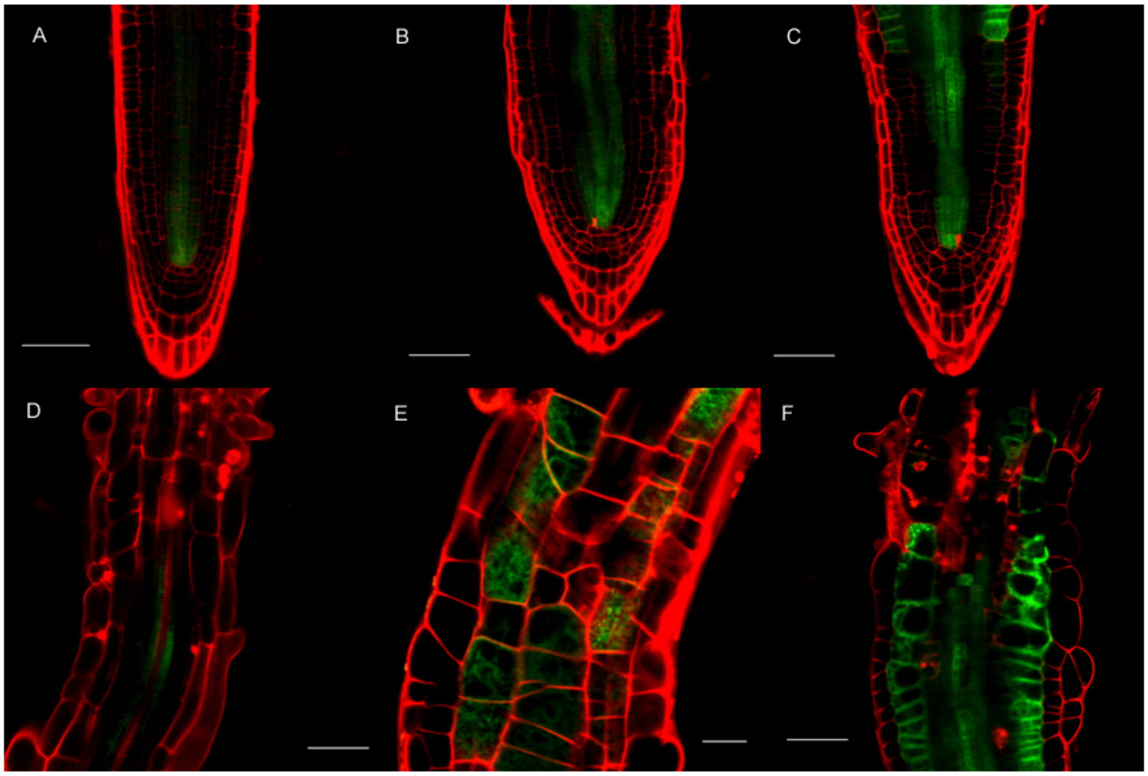

Figure 3. Auxin- and gibberellic acid-inducible expression of *At4g32460.* Transgenic 5-day-old seedlings were treated for 0 h (A, D) or 24 h with 2 μM GA (B,E) or for 48 h with 2 μM indole-3-acetic acid (IAA) (C,F). A) GFP fluorescence in provascular tissue of meristematic zone in primary roots (as in Figure 2). B) GFP fluorescence in provascular and vascular tissue of meristematic and transition zone in primary roots. C) GFP fluorescence in provascular and vascular tissue of meristematic and transition zone in primary roots. GFP fluorescence was also detected in cortical cells of transition zone. D) GFP fluorescence in pericycle cells of vascular tissue of maturation zone in primary roots (as in Figure 2). E) GFP fluorescence in vascular tissue and cortical cells of maturation

zone in primary roots. F) GFP fluorescence in vascular tissue and cortical cells of maturation zone in primary roots.

4. Negative control for germination assays.

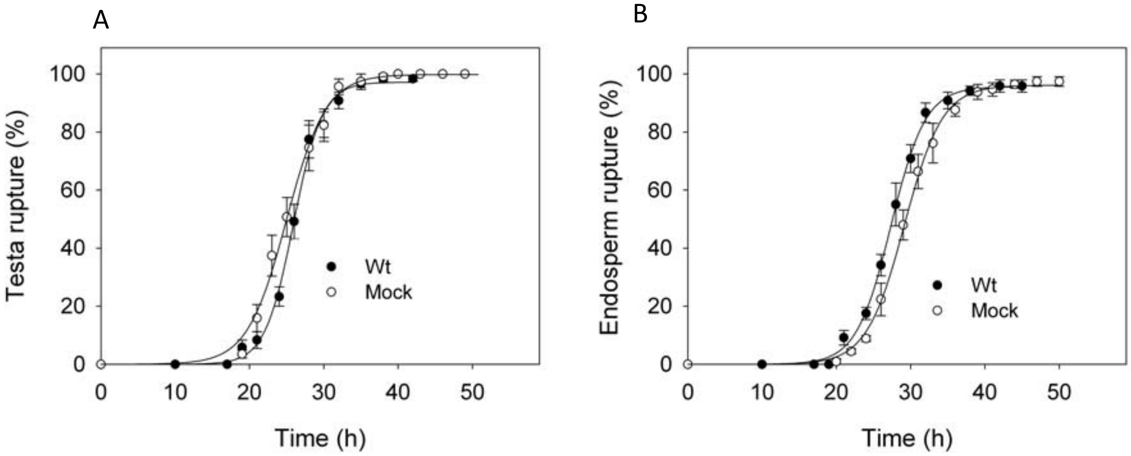

Figure 4. Effect of empty plasmid on germination performance. A) Cumulative testa rupture curve. B) Cumulative endosperm rupture curve. Germination assays were carried out in triplicate at 20 °C.

5. Mucilage release in water imbibed OE lines

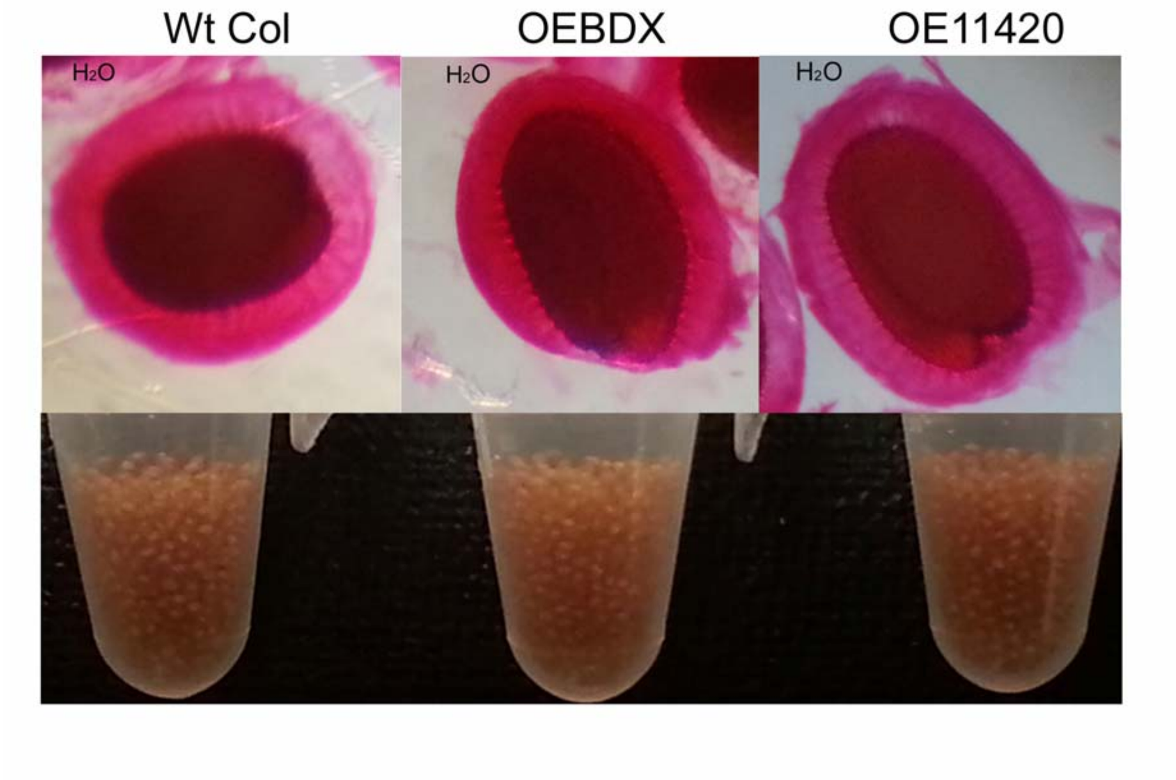

Figure 5. OEBDX and OE11420 seeds did not show defective mucilage release after imbibition in water. Dry seeds (10 mg) were hydrated (43) and stained with ruthenium red to detect pectin (upper panel).

6. Phenotypic analyses of T-DNA *bdx-2*

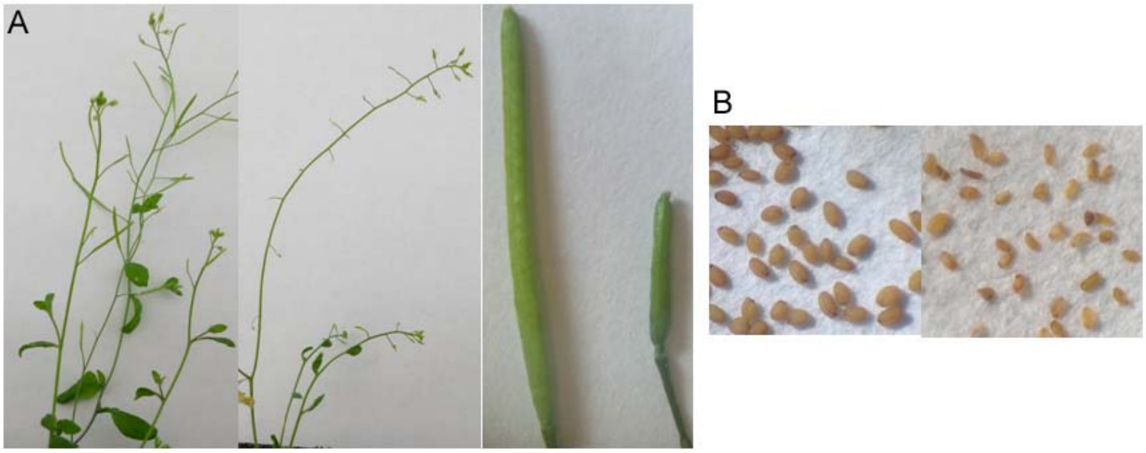

Figure 6. Phenotypic analyses of T-DNA *bdx-2*. A) Phenotypic comparison between heterozygous *bdx-2* plant and wt (control). Siliques of heterozygous *bdx-2* plants were shorter than those of wt. B) Many seeds from *bdx-2* plants were small and wrinkled (right).
